# Addressing Individual Layers and Their Optical Properties in Artificial MoS_2_ Bilayers via Sulfur Isotope Labeling

**DOI:** 10.1021/acs.jpcc.4c03132

**Published:** 2024-07-23

**Authors:** Antonin Kralik, Golam Haider, Vaibhav Varade, Martin Kalbac, Jana Vejpravova

**Affiliations:** † Heyrovsky Institute of Physical Chemistry of the CAS, v.v.i., Dolejskova 3, Prague 8 CZ-182 23, Czech Republic; ‡ Department of Condensed Matter Physics, Faculty of Mathematics and Physics, Charles University, Ke Karlovu 5, Prague 2 CZ-121 16, Czech Republic

## Abstract

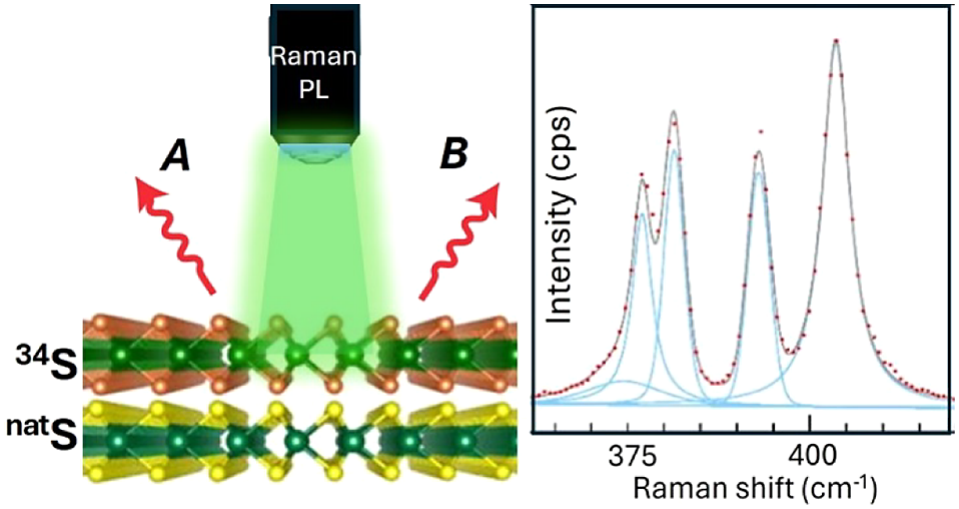

The physicochemical properties of van der Waals (vdW) heterostructures are driven by the delicate interactions between the individual layers in a multilayer stack. While addressing the monolayers of different compositions in the multilayer is feasible, exploring the intrinsic properties of the monolayers of the same composition within a multilayer is extremely challenging. This becomes of utmost importance in energy conversion and storage concepts based on layered vdW materials. For example, the charge distribution on the individual layers can be determined, and the behavior can be disentangled. We introduce sulfur isotope labeling as a powerful tool for separately addressing monolayers in vdW heterostructures composed of transition metal disulfides. Using chemical vapor deposition (CVD), we prepared monolayers of MoS_2_ using natural sulfur (^Nat^S) and ^34^sulfur (^34^S) as precursors. Artificial bilayers were then prepared by transferring Mo^34^S_2_ onto Mo^Nat^S_2_. Thanks to the different masses of ^Nat^S and ^34^S, we were able to disentangle the spectral fingerprints of phonons and excitons in the two layers using Raman and photoluminescence microspectroscopy. Also, the charge distribution on the individual layers was revealed through Raman spectra analysis. Our work thus provides a different perspective for understanding the functionalities and optical properties of smart architecture based on transition metal chalcogenides.

## 1 Introduction

The discovery of graphene as a stable two-dimensional (2D) material with unique physical and chemical properties, as well as a wide variety of applications,^1−3^ has sparked the discovery and extensive research of other materials capable of forming nearly atomically thin sheets with remarkable electronic properties. Arguably, the most important group of these materials is transition metal dichalcogenides (TMDs). These compounds have the general formula MX_2_, where M is a transitional metal and X is a chalcogen. They can be either semiconducting or metallic. In the case of semiconducting TMDs, the most notable feature is the transition from an indirect bandgap in bulk material to a direct bandgap in mono layers, predetermining them as promising candidates for various optoelectronic concepts.^4−6^ Furthermore, their competitive physio-chemical properties predestine them as significant materials for gas- and biosensors, piezoelectric devices, photonic devices, and energy storage.^7^ The properties of TMDs are easily reformed by strain^8^ and exhibit strong light–matter interactions.^9^

Isotopic effects are traditionally studied in bulk semiconductors, as they are important for thermal management and fine-tuning their optical properties. Nevertheless, research on isotopic effects in 2D materials remains relatively restricted. Most studies have focused on graphene, where isotope engineering has allowed for the disentangling of strain and/or doping in individual layers of multilayered graphene and has provided insights into the growth mechanisms of bilayer and multilayer graphene.^10,11^ It has been also demonstrated that introducing different isotopes is extremely useful for spectroscopic studies of individual monolayers in graphene.^11^ Temperature-dependent experiments on hBN, investigating the shear and breathing motions of adjacent layers, highlighted the unique impact of isotope engineering on vdW interactions in layered materials, offering insights into the understanding and control of vdW bonding in such materials.^12^ Other reports have studied the effects of isotopes in hBN on phonon lifetime^13^ and Raman response of hBN under high pressure.^14^

In the work of Li et al., tuning of the lattice phonons using isotopes has been reported for 2D MoS_2_ with ^92^ Mo and ^100^ Mo isotope enrichment for the first time.^15^ Compared with materials containing a natural mixture of Mo isotopes, isotopically pure samples showed higher thermal conductivity, stronger photoluminescence, and longer exciton lifetime. Besides the moderate changes in optical and thermal properties, the authors also reported some changes in the Raman spectra. Since molybdenum has seven naturally occurring stable isotopes with relatively similar abundances and an average atomic mass of around 96, the observed changes were relatively small. Hence, using Mo isotopes to label the MoS_2_ layers is not practical.

Interestingly, it has also been demonstrated that isotopes can affect optical properties. Although this represents a potentially elegant way to tailor the properties of 2D materials, there are very limited reports addressing this aspect. One example is the work of Wu et al., which demonstrated changes in optical properties (bandgap) when comparing isotopically pure^186^W^80^Se_2_ and ^Nat^W^Nat^Se_2_.^16^ Recently, it was also found that isotopes affect the optical bandgap of MoS_2_.^17^ This observed trend, contrary to that seen in conventional semiconductors, is elucidated through many-body perturbation and time-dependent density functional theories, revealing significant exciton binding energy renormalizations, surpassing ground-state renormalization energies, owing to the robust coupling between the confined excitons and phonons.^17^

The approach of introducing different isotopes in TMDs is somewhat limited by the common methods of TMD preparation, such as physical^18,19^ or chemical exfoliation,^20−22^ and CVD.^23−25^

Until now, sulfur isotope engineering has been limited to a single study on monolayer MoS_2_.^26^ In this pioneering work, we succeeded in preparing isotopically engineered MoS_2_ monolayers with ^32^S, ^34^S, 50:50 ^32^S:^34^S, and ^Nat^S. The availability of isotopically defined monolayers enabled us to disentangle the crucial role of phonons in the optoelectronic properties down to low temperatures. Since sulfur naturally consists of 95% of ^32^S isotope, 4.25% of ^34^S isotope, and traces of other isotopes, sulfur isotope engineering provides better insight into the lattice dynamics and optical response of bilayers (and multilayers) through a combination of layer-selective vibrational spectroscopy and photoluminescence studies.

The heterostructures of 2D materials are widely studied because they enable the bottom-up synthesis of materials with tailored properties. Normally, heterostructures are made from different materials. However, in this case, the crystal lattices do not perfectly match, which creates another degree of freedom. On the other contrary, isotope engineering can offer a unique opportunity to study the interaction of the two layers with the same lattice parameters.

In this work, we demonstrate the successful preparation of isotopically resolved mixed bilayers of Mo^34^S_2_ and natural Mo^Nat^S_2_ (containing mostly^32^S) to study the mutual interaction of the layers in the heterostructure. The artificial bilayers were prepared using PMMA-assisted transfer of CVD-grown monolayers. The mono and bilayers were characterized using atomic force microscopy (AFM), Raman and photoluminescence (PL) microspectroscopies, and time-resolved PL spectroscopy. We demonstrate that sulfur isotope labeling is a powerful tool for addressing individual layers in TMDs and their vdW heterostructures using spectroscopic techniques. Also, isotope engineering enables the separate tailoring of lattice and exciton dynamics in the constituent monolayers.

## 2 Experimental Section

### 2.1 Synthesis of MoS_2_ Monolayers

MoO_2_ (15 mg) was placed in a quartz crucible (L × W × D 40 × 10 × 2 mm^3^). Silicon thermal oxide wafer (525 μm of Si with 300 nm of SiO_2_; L × W 30 × 12 mm^2^) was cleaned by successive sonication in DI water, acetone, and isopropanol, and DI solution of PTAS (1 mM) was spin-coated on its surface. The wafer was then placed face down on top of the crucible, which was subsequently inserted into the middle of a quartz tube (400 × 15 mm). Three pieces of sulfur (totaling 25 mg) were placed in tubes positioned 14, 16, and 18 cm from the crucible, respectively. These tubes were then inserted into a larger quartz tube (800 × 25 mm^2^). The larger tube was connected to an argon gas line at one end and to a bubbler filled with a 100 mM aqueous solution of KOH at the other end. The tube was flushed with argon for 15 min. Following this, the section of the tube containing the crucible was heated under a constant flow of argon (120 cm^3^/min) in a cylindrical furnace, with the temperature increasing at a rate of 40 °C per minute. When the temperature reached 785 °C, the tube was shifted to introduce the sulfur into the furnace. Once the temperature reached 835 °C, it was maintained at this level for 10 min, after which the furnace was opened and the system was allowed to cool down.

### 2.2 Transfer of MoS_2_

A solution of PMMA (toluene; 6 wt %) was spin-coated (2500 rpm for 30 s) onto the surface of a Si/SiO_2_ wafer containing as-grown MoS_2_. After drying, the edges of the wafer were broken off, and the wafer was placed in a 1 M aqueous solution of KOH. After etching off the SiO_2_ layer, the polymer containing MoS_2_ remained on the surface from which it was transferred onto three subsequent cleaning baths with DI water using a clean piece of Si/SiO_2_ wafer treated with oxygen plasma. After washing, the polymer was transferred onto a mixture of DI water and isopropanol (25 vol % of IPA). From this bath, it was collected onto the surface of a Si/SiO_2_ wafer containing another as-grown MoS_2_ and dried in a stream of argon. Finally, PMMA was washed off by using warm (60 °C) acetone.

### 2.3 Microscopic and Spectroscopic Characterization

Ambient, room-temperature Raman and PL spectral maps were measured using a WITec Alpha300R spectrometer equipped with a piezo stage and a RayShield Coupler. The measurements were performed with 532 nm (2.33 eV) or 633 nm (1.96 eV) laser excitation, at a laser power of approximately 1 mW for Raman spectra and 10 μW for PL, and a grating of 600 or 1800 lines/mm for the PL or Raman spectra, respectively. The Raman spectrometer was calibrated using the Si line at 520.2 cm^–1^. The Raman and PL peaks were fitted using a pseudo-Voigt profile to account for spectral parameter variations within the laser spot area.

AFM was measured using Bruker AFM Dimension Icon in PeakForce QNM mode with ScanAsyst and the data were processed in Gwyddion software.^27^

Time-resolved PL (TRPL) measurements were performed on an Olympus FluoView1000 confocal system coupled to a PMT detector (tau-SPAD, Picoquant) with subnanosecond TCSPC capability (HydraHarp 400, Picoquant). The samples were excited with a 420 nm laser (2.95 eV; LDH-D-C-420, Picoquant) with 0.5 nW μm^–2^ power, a frequency of 40 MHz, and a pulse duration of 80 ps.

## 3 Results and Discussion

### 3.1 Sample Preparation

Isotopically labeled MoS_2_ monolayers were prepared via CVD growth on Si/SiO_2_ substrates. To form an MoS_2_ bilayer, a monolayer of MoS_2_ was transferred on top of another as-grown monolayer using the PMMA-assisted wet transfer method (described in the Experimental section). We have recently demonstrated that the properties of MoS_2_ monolayers change significantly after transfer compared to as-grown monolayers.^28^ However, the original properties can be largely recovered through annealing.^28^ Therefore, in the following paragraphs, we discuss the properties of the annealed samples to minimize the impact of the transfer process on the bilayer properties.

### 3.2 Optical and AFM Imaging

**Figure 1:**
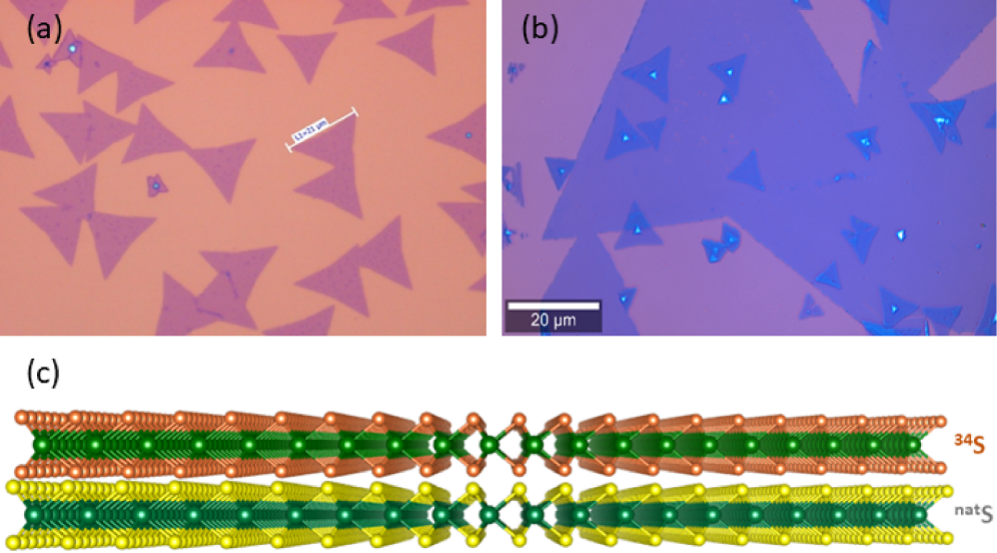
Optical images of CVD-grown monolayer of Mo^34^S_2_ (a) and Mo^34^S_2_/Mo^Nat^S_2_ bilayer prepared via wet transfer (b). Panel (c) shows a schematic of the bilayer stacking.

Figure 1a shows an optical image of typical MoS_2_ triangles obtained by CVD growth. The growth process leads to samples with varying size distributions of the crystallites and different densities of crystal formation depending on the location. Nevertheless, nearly all the samples were monolayers, regardless of the isotope content. The thickness of the prepared “crystals” of MoS_2_ revealed by AFM was found to be around 0.7 nm, which confirms the presence of monolayers (a typical AFM topography image is shown in Figure S1). Figure 1b represents an optical image of a Mo^34^S_2_/Mo^Nat^S_2_ bilayer (the scheme of the stacking is given in Figure 1c). As it is apparent from Figure 1b, the sample contains three distinct regions—monolayer Mo^Nat^S_2_, monolayer Mo^34^S_2_, and bilayer Mo^34^S_2_/Mo^Nat^S_2_, which are expected to reveal different fingerprints in Raman and PL spectra.

### 3.3 Raman Spectroscopy and PL

**Figure 2:**
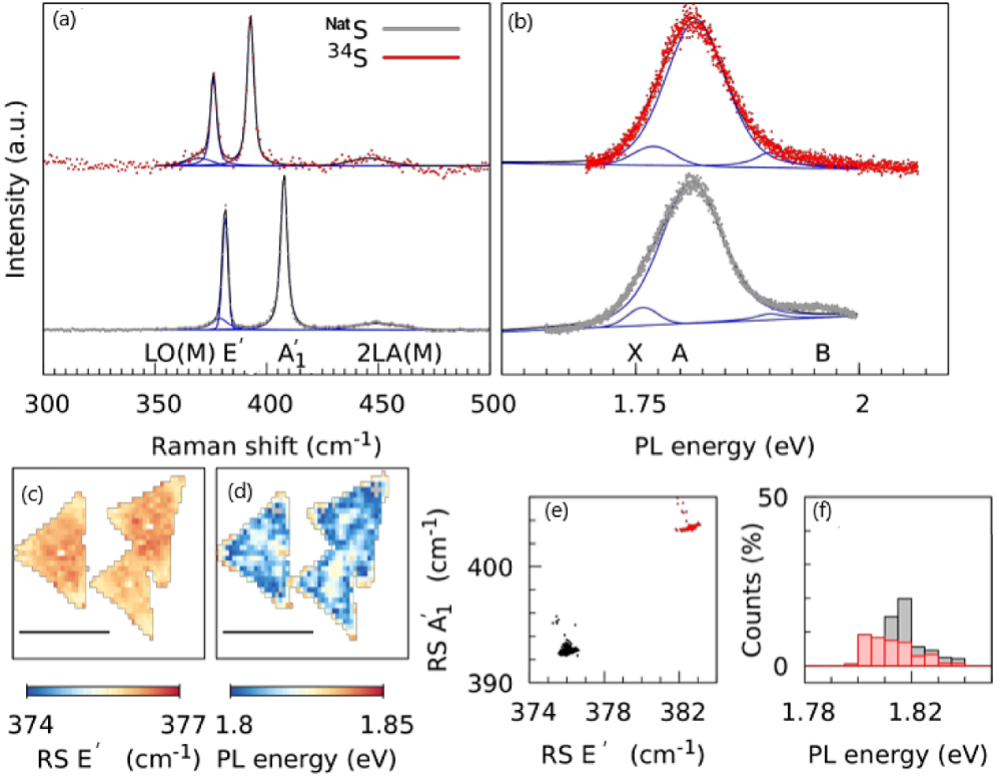
(a) Raman spectroscopic characterization of Mo^Nat^S_2_ (red) and Mo^34^S_2_ (gray) monolayers obtained using 532 nm laser excitation, (b) PL spectra of Mo^Nat^S_2_ (red) and Mo^34^S_2_ (gray), together with the fit of trion (X), A exciton (A) and B exciton (B), (c) Raman spectral maps of the shift of the E’ mode, and (d) the energy of the A exciton in Mo^34^S_2_. (e) Correlation between Raman shifts of E’ and A’_1_ modes of Mo^Nat^S_2_ (gray) and Mo^34^S_2_ (red) monolayers. (f) Histograms are shown to compare the energy of A exciton between Mo^Nat^S_2_ and Mo^34^S_2_ monolayers.

Figure 2 shows the out-of-resonance spectroscopic comparison between the as-grown monolayers of Mo^Nat^S_2_ and Mo^34^S_2_ under 532 nm laser irradiation. There is a significant change in shifts of E’ and A’_1_ modes caused by the presence of heavier sulfur isotope. E’ shifts from 382 to 377 cm^–1^, while A’_1_ shifts from 409 to 396 cm^–1^. The observed shift can be explained by the decrease in phonon frequencies caused by the higher mass of ^34^S, which is a well-known effect for isotopically labeled compounds.^29^

The spatial distribution of the important spectral features, i.e., the Raman shift (RS) map of the E’ and the PL energy map of the A exciton, is shown in Figure 2,c,d, respectively. The large sets of spectral parameters derived from the Raman and the PL maps enable to get more information on doping, strain, and defects in TMDs.^28,30^

It has been shown by Papagelis and coworkers^30^ that the correlation of the spectral parameters of the A’_1_ and E’ modes provides valuable information about variations of strain (ε) and doping (*n*) in MoS_2_ monolayers. By plotting the RS of the E’ mode against that of the A’_1_ mode, the information about doping and strain can be extracted, assuming that strain and doping are decoupled. A simplified ε-n correlation diagram for Mo^Nat^S_2_ and Mo^34^S_2_ is shown in Figure 2e. The origin of the correlation plot representing a “pristine” MoS_2_ monolayer with natural isotopic abundance was reported to be (384.5 cm^–1^ for [RS E’], 402.5 cm^–1^ for [RS A’_1_]). The cloud of E’ – A’_1_ correlation pairs plotted for Mo^Nat^S_2_ (red points) is centered around (384 cm^–1^ for [RS E’], 403 cm^–1^ for[RS A’_1_]) , indicating moderate expansion (ε = 0.15%) and n-type doping (*n* = −0.15 × 10^13^ cm^–2^). The correlation pairs for Mo^34^S_2_ (gray points) are downshifted to (376 cm^–1^ for [RS E’], 393 cm^–1^ for [RS A’_1_]) as the Raman spectral features are located at lower wavenumbers in agreement with the larger isotopic mass of ^34^S. Considering that both types of monolayers were grown using the identical approach, we estimate the “zero point” for pristine Mo^34^S_2_ to be around (375.5 cm^–1^ for [RS E’], 392.5 cm^–1^ for [RS A’_1_]).

Finally, Raman spectra were recorded on Mo^Nat^S_2_ and Mo^34^S_2_ monolayers by using a 633 nm laser (shown in Figure S2). Since the 633 nm laser matches the energy of the optical transition, the Raman spectrum is resonant, allowing the observation of several combinatorial modes. These modes include a combination of an A’_1_ mode and a longitudinal acoustic (LA) mode at the M point of the Brillouin zone (178 cm^–1^ in Mo^Nat^S_2_, 171 cm^–1^ in Mo^34^S_2_),^31^ and a so-called “b” mode involving a polariton (416 cm^–1^ in Mo^Nat^S_2_, 413 cm^–1^ in Mo^34^S_2_).^32,33^ In agreement with the out-of-resonance Raman spectra, all Raman modes show significant shifts when Mo^Nat^S_2_ (a) and Mo^34^S_2_ (b) are compared.

Additionally, there is a clear distinction in the PL spectra between Mo^Nat^S_2_ and Mo^34^S_2_. The PL spectrum of Mo^Nat^S_2_ is dominated by a broad band ranging from 1.68 and 1.92 eV, which consists of several contributions. As previously demonstrated,^28^ the main components of this band are the free trion and exciton. The existence of the so-called bound trion has also been reported. This quasiparticle appears between the free trion and exciton band and can be observed in doped samples.^34^Figure 2a (right) shows the decomposition of the PL peak into three bands assigned to the free trion, bound trion, and exciton. The sum of the fitted bands is centered at 1.81(7) eV. The PL spectrum of Mo^34^S_2_ is shifted toward higher energies and the PL band is centered at about 1.83(6) eV. To demonstrate the significance of the PL shift caused by the isotopic exchange, histograms of the A exciton energies for the Mo^Nat^S_2_ and Mo^34^S_2_ are presented in Figure 2f. The shift of the A exciton toward higher energies with increasing mass of the isotope is in agreement with trends observed in bulk semiconductors. However, this behavior contrasts with recent observations for isotopically pure ^100^ MoS_2_ and ^92^ MoS_2_. The distinction arises from the influence of specific elements on the phonon branches. The molybdenum isotopes primarily impact acoustic phonon branches,^17^ while sulfur isotopes affect high-energy optical phonon branches.^26^ This variation plays a role in modulating exciton energy renormalization.

In addition, the PL band of Mo^34^S_2_ is narrower and approximately 1.8 times more intense (based on the area under the curve, AUC) compared to the PL band of Mo^Nat^S_2_. The increased intensity can be understood by the fact that Mo^Nat^S_2_ contains about 5% of other isotopes (^33^S: 0.8%; ^34^S: 4.2%; ^36^S: 0.02%), while Mo^34^S_2_ contains 99.91% of ^34^S. The isotope impurities generally reduce Pl lifetimes, leading to decreased PL intensity and a broader spectral response.^26,29^

**Figure 3:**
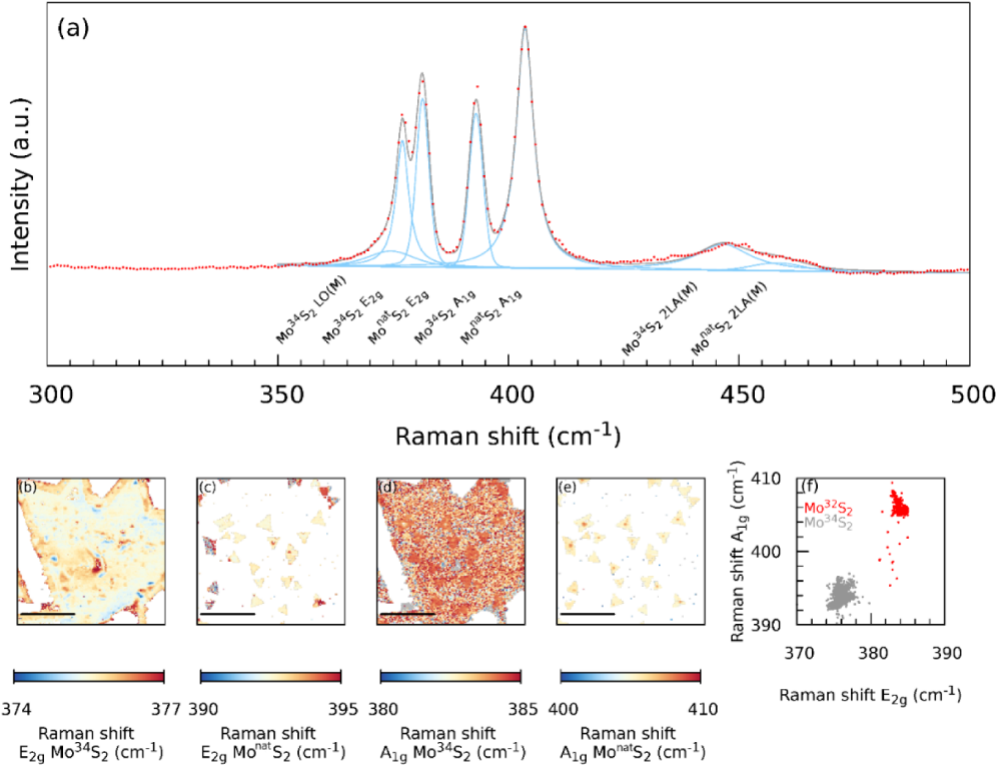
Raman spectroscopic characterization of a Mo^34^S_2_/Mo^Nat^S_2_ bilayer using 532 nm laser excitation energy. (a). Raman spectral maps of the shift of the E_2g_ and A_1g_ modes of the bilayer (b–e). Correlation between Raman shifts of E_2g_ and A_1g_ modes of Mo^Nat^S2 (gray) and Mo^34^S_2_ (red) monolayer (f).

Out-of-resonance spectra of the artificial Mo^34^S_2_/Mo^Nat^S_2_ bilayer obtained under 532 nm laser irradiation are shown in Figure 3a. The spectra are clearly a superposition of E’ and A’_1_ modes of Mo^34^S_2_ and Mo^Nat^S_2_ monolayers, both in terms of RS and intensity. This observation suggests that the phonons of the individual layers do not interact significantly with each other. Please note that we use the notation of Raman bands for bilayer MoS_2_ (A_1g_, E_2g_) when discussing the artificial Mo^34^S_2_/Mo^Nat^S_2_ bilayer.

The spatial distribution of the RS in the top (Mo^34^S_2_) and bottom (Mo^Nat^S_2_) layers are shown in Figure 3,b–e. The Raman imaging is in excellent agreement with the optical and AFM microscopic observations. The top layer (Mo^34^S_2_) extends over a large area of the sample, as suggested by the microscopy images, while the features of the bottom layer are located at the triangle-shaped areas corresponding to the small flakes of Mo^Nat^S_2_. The artificial Mo^34^S_2_/Mo^Nat^S_2_ bilayers can be quite easily identified in the RS map of the A_1g_ (A’_1_) corresponding to the top layer (Figure 3d) as the areas of the increased RS match the position of the small triangle-like flakes of the bottom layer (Mo^Nat^S_2_). The increase in the RS can be attributed to moderate compression acting on the top layer. A less evident but clear difference is observed in the map of the RS of the E_2g_ (E’) mode of the bottom layer. The RS values in the flakes located completely outside the top layer area show significantly lower values.

To get some estimate of the strain and doping acting in both layers in the Mo^34^S_2_/Mo^Nat^S_2_ bilayer, the ε-n correlation diagram was plotted as described for the monolayers previously (Figure 3f). Compared to the isolated monolayers, the correlation points are clearly extended over a larger area of the diagram, with the center of both clouds shifting toward larger negative doping and moderate tensile strain.

**Figure 4:**
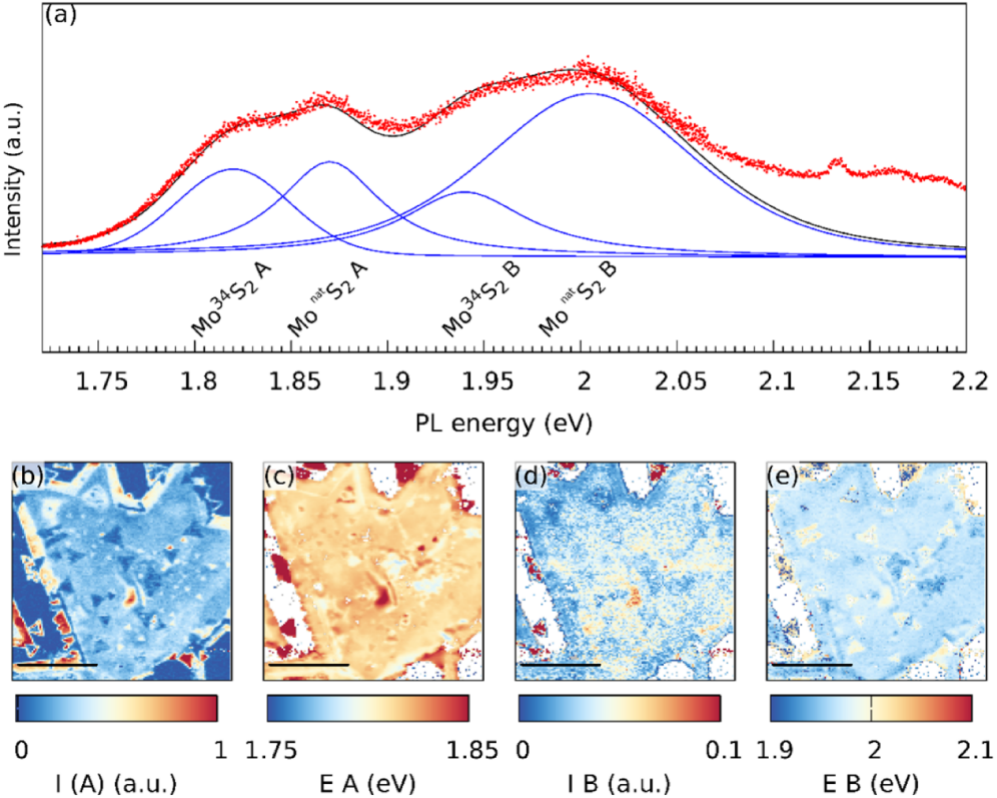
PL spectroscopic characterization of the Mo^34^S_2_/Mo^Nat^S_2_ bilayer with depicted excitons A and B (a). Spectral maps of intensity I and energy E for the A and B excitons (b–e).

While the appearance and analysis of the Raman spectra are quite straightforward, the PL spectra of the artificial MoS_2_ bilayer (shown in Figure 4) are strongly quenched; the PL intensity is about 14 times lower (AUC comparison) than that of the Mo^Nat^S_2_ single layer. The PL peak is centered at about 1.82(5) eV, which is somewhat between the PL peaks of the Mo^Nat^S_2_ and Mo^34^S_2_ monolayers (Figure S3). In the oriented MoS_2_ bilayer, quenching of the signal is expected because of the direct-to-indirect bandgap transition.^28^ In the case of the artificial isotopically resolved bilayer, the quenched PL may also suggest that the MoS_2_ layers are electronically coupled. However, this observation somewhat contrasts with the conclusions drawn from the above-mentioned Raman spectra, which reveal that phonons of the individual layers do not interact significantly, suggesting a random orientation of the two layers.^36,37^ Thus, this inconsistency can be better understood by considering the tightness of the contact and the overall impact of the transfer procedure on the flatness and purity of the layers, respectively. The interaction of phonons, which are strongly localized in individual layers, requires both a very tight contact and proper mutual orientation of the layers. In our case, the MoS_2_ bilayer was prepared by a subsequent transfer of the CVD grown layers. As a result, some residuals of impurities may be trapped on top of and between the layers. Also, nanoscale corrugations occur due to the polymer-assisted transfer procedure.^28^ Consequently, the PL spectra reveal a suppression of the signal, while the Raman spectra featuring the fingerprints of the individual layers are less affected.

**Figure 5:**
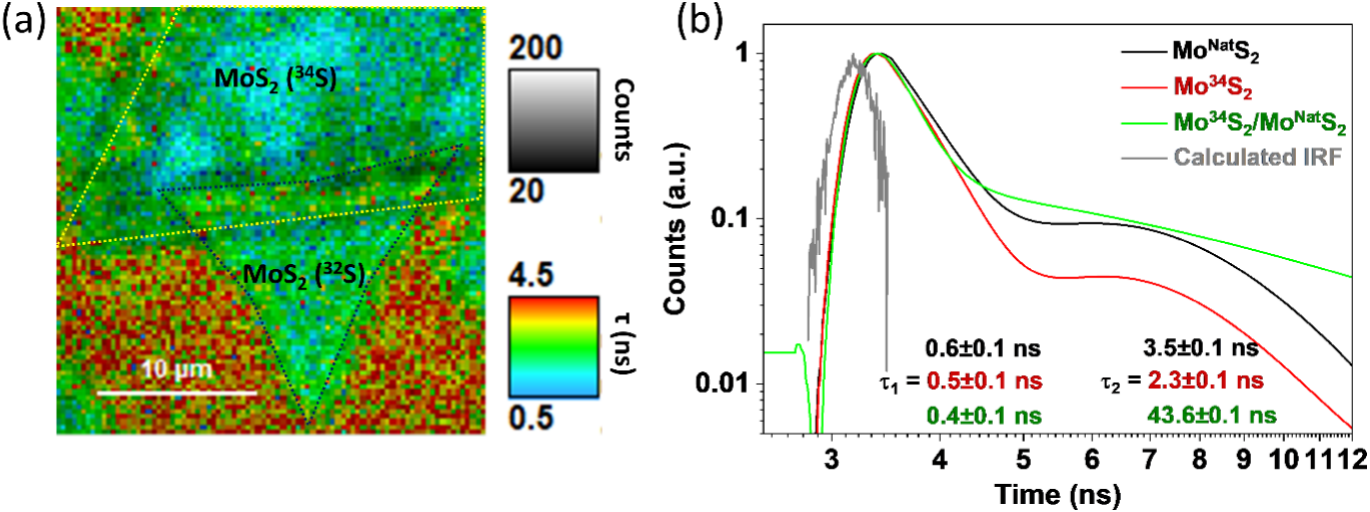
Time-resolved PL spectroscopic analysis. Spatially resolved TRPL, where a position containing individual monolayers of different isotopes and their heterostructure, has been chosen as the region of interest (a). The yellow and black dotted lines serve as guides to different layers. The transient decay profile of the layers, the heterostructure, and the instrument response function (IRF) of the measurement system (b).

In order to gain more insight into the isotope-induced PL variation and the exciton dynamics at the monolayers and the heterostructure, we performed time-resolved PL spectroscopy measurements. The obtained decay profiles of both as-grown monolayers and the Mo^34^S_2_/Mo^Nat^S_2_ bilayer are shown in Figure 5. The sample area containing individual monolayers and heterostructures was chosen as the region of interest for comparison. The spatial distribution of lifetime over the flakes and the heterostructure is shown in Figure 5a. The overall lifetimes for the individual layers were found to be comparable for both the fast (τ_1_) and slower (τ_2_) channels of the as-grown Mo^Nat^S_2_ and Mo^34^S_2_.

### 3.4 Time-Resolved PL (TRPL)

The decay constants obtained for monolayer samples in Figure 5b are comparable to the values reported in our earlier work by Varade et al.,^26^ where significant variations from the reported decay profile are related to the transfer-induced change in the optical properties of the layers as observed previously.^28^ However, the decay curve at the heterostructure is significantly different than the monolayers resembling a faster τ_1_ and slower τ_2_. This is attributed to the homojunction formation; though the layers are isotopically distinct, the bandgap remains the same.

We note that our data represent averages of different mutual orientations of the MoS_2_ layers. It is known that a particular mutual orientation can influence the optical properties.^35^ Conversely, the impact of stacking on the Raman spectra is relatively weak, as shown in the same study. Therefore, the conclusions about strain and doping in the individual layers derived from the Raman data are not affected, and in fact, one can consider studying how the strain and doping are influenced by the twist angle. The effect of the isotope on the exciton energy is significantly stronger than the effect of the twist angle. It is also known that the twist angle may vary slightly within a bilayer. This can actually explain the small variation in the optical properties observed in the PL maps.

## 4 Conclusions

We successfully prepared artificial bilayers containing Mo^34^S_2_ and Mo^Nat^S_2_ via PMMA-assisted transfer of the as-grown MoS_2_ monolayers. The presence of heavier sulfur atoms caused a clear decrease in the Raman shift of the principal Raman active modes in both resonance and out-of-resonance experiments, as well as higher PL intensity and shorter overall PL lifetimes, which is following theoretical predictions and recent results obtained on isotopically pure monolayers. However, analyses of the optical experiments conducted on the artificial bilayers showed mixed results. Significant PL quenching suggests that even the transferred layers are electronically coupled. On the contrary, Raman spectra featuring a superposition of the spectra typical for the isolated monolayers suggest that the phonon coupling is not significant and the moderate variations are rather caused by the doping and strain effects estimated from the ε-n correlation diagrams. We would like to point out that we successfully tested the CVD growth of W^34^S_2_ under the same protocol (optical images and spectroscopic characterizations are shown in Figure S4), which demonstrates the generality and great potential of the isotope labeling in TMD research. Thus, the concept of isotopically labeled TMD bilayers and multilayers can be utilized in studies of electronic interactions, strain, doping, and defect formation, allowing these phenomena to be examined independently for each layer within a single spectroscopic experiment.

## Supporting Information Available

AFM image of the fabricated heterostructure, Raman spectra of natural and ^34^S isotopically labeled MoS_2_ using a 632 nm laser, and optical characterization of CVD-grown WS_2_ with ^34^S isotopic labeling, featuring an optical image, Raman spectra (532 nm laser), PL spectrum, and PL mapping (PDF)

## References

[ref1] Bonaccorso F.; Sun Z.; Hasan T.; Ferrari A. C. Graphene photonics and optoelectronics. Nat. Photonics 2010, 4, 611–622. 10.1038/nphoton.2010.186.

[ref2] Castro Neto A. H.; Guinea F.; Peres N. M. R.; Novoselov K. S.; Geim A. K. The electronic properties of graphene. Rev. Mod. Phys. 2009, 81, 109–162. 10.1103/RevModPhys.81.109.

[ref3] Zhu Y.; Murali S.; Cai W.; Li X.; Suk J. W.; Potts J. R.; Ruoff R. S. Graphene and Graphene Oxide: Synthesis, Properties, and Applications. Adv. Mater. 2010, 22, 3906–3924. 10.1002/adma.201001068. 20706983

[ref4] Butler S. Z.; Hollen S. M.; Cao L.; Cui Y.; Gupta J. A.; Gutiérrez H. R.; Heinz T. F.; Hong S. S.; Huang J.; Ismach; et al. Progress, Challenges, and Opportunities in Two-Dimensional Materials Beyond Graphene. ACS Nano 2013, 7, 2898–2926. 10.1021/nn400280c. 23464873

[ref5] Duan X.; Wang C.; Pan A.; Yu R.; Duan X. Two-dimensional transition metal dichalcogenides as atomically thin semiconductors: Opportunities and challenges. Chem. Soc. Rev. 2015, 44, 8859–8876. 10.1039/C5CS00507H. 26479493

[ref6] Jariwala D.; Sangwan V. K.; Lauhon L. J.; Marks T. J.; Hersam M. C. Emerging Device Applications for Semiconducting Two-Dimensional Transition Metal Dichalcogenides. ACS Nano 2014, 8, 1102–1120. 10.1021/nn500064s. 24476095

[ref7] Choi W.; Choudhary N.; Han G. H.; Park J.; Akinwande D.; Lee Y. H. Recent development of two-dimensional transition metal dichalcogenides and their applications. Mater. Today 2017, 20, 116–130. 10.1016/j.mattod.2016.10.002.

[ref8] Shi H.; Pan H.; Zhang Y.-W.; Yakobson B. I. Strong ferromagnetism in hydrogenated monolayer MoS_2_ tuned by strain. Phys. Rev. B 2013, 88, 205305 10.1103/PhysRevB.88.205305.

[ref9] Britnell L.; Ribeiro R. M.; Eckmann A.; Jalil R.; Belle B. D.; Mishchenko A.; Kim Y. J.; Gorbachev R. V.; Georgiou T.; Morozov; et al. Strong Light-Matter Interactions in Heterostructures of Atomically Thin Films. Science 2013, 340, 1311 10.1126/science.1235547. 23641062

[ref10] Ek-Weis J.; Costa S.; Frank O.; Kalbac M. Heating Isotopically Labelled Bernal Stacked Graphene: A Raman Spectroscopy Study. J. Phys. Chem. Lett. 2014, 5, 549–554. 10.1021/jz402681n. 26276607

[ref11] Frank O.; Kavan L.; Kalbac M. Carbon isotope labelling in graphene research. Nanoscale 2014, 6, 6363–6370. 10.1039/c4nr01257g. 24817019

[ref12] Vuong T. Q. P.; Liu S.; Van der Lee A.; Cuscó R.; Artús L.; Michel T.; Valvin P.; Edgar J. H.; Cassabois G.; Gil B. Isotope engineering of van der Waals interactions in hexagonal boron nitride. Nat. Mater. 2018, 17, 152–158. 10.1038/nmat5048. 29251722

[ref13] Cuscó R.; Edgar J. H.; Liu S.; Li J.; Artús L. Isotopic Disorder: The Prevailing Mechanism in Limiting the Phonon Lifetime in Hexagonal BN. Phys. Rev. Lett. 2020, 124, 167402 10.1103/PhysRevLett.124.167402. 32383900

[ref14] Cuscó R.; Pellicer-Porres J.; Edgar J. H.; Li J.; Segura A.; Artús L. Phonons of hexagonal BN under pressure: Effects of isotopic composition. Phys. Rev. B 2021, 103, 085204 10.1103/PhysRevB.103.085204.

[ref15] Li X.; Zhang J.; Puretzky A. A.; Yoshimura A.; Sang X.; Cui Q.; Li Y.; Liang L.; Ghosh A. W.; Zhao H. Isotope-Engineering the Thermal Conductivity of Two-Dimensional MoS_2_. ACS Nano 2019, 13, 2481–2489. 10.1021/acsnano.8b09448. 30673215

[ref16] Wu W.; Morales-Acosta M. D.; Wang Y.; Pettes M. T. Isotope Effect in Bilayer WSe_2_. Nano Lett. 2019, 19, 1527–1533. 10.1021/acs.nanolett.8b04269. 30753084

[ref17] Yu Y.; Turkowski V.; Hachtel J. A.; Puretzky A. A.; Ievlev A. V.; Din N. U.; Harris S. B.; Iyer V.; Rouleau C. M.; Rahman T. S. Anomalous isotope effect on the optical bandgap in a monolayer transition metal dichalcogenide semiconductor. Sci. Adv. 2024, 10, eadj0758 10.1126/sciadv.adj0758. 38381831 PMC10881028

[ref18] Novoselov K. S.; Jiang D.; Schedin F.; Booth T. J.; Khotkevich V. V.; Morozov S. V.; Geim A. K. Two-dimensional atomic crystals. Proc. Natl. Acad. Sci. U. S. A. 2005, 102, 10451 10.1073/pnas.0502848102. 16027370 PMC1180777

[ref19] Late D. J.; Liu B.; Matte H. S. S. R.; Rao C. N. R.; Dravid V. P. Rapid Characterization of Ultrathin Layers of Chalcogenides on SiO_2_/Si Substrates. Adv. Funct. Mater. 2012, 22, 1894–1905. 10.1002/adfm.201102913.

[ref20] Nicolosi V.; Chhowalla M.; Kanatzidis M. G.; Strano M. S.; Coleman J. N. Liquid Exfoliation of Layered Materials. Science 2013, 340, 1226419 10.1126/science.1226419.

[ref21] Zeng Z.; Sun T.; Zhu J.; Huang X.; Yin Z.; Lu G.; Fan Z.; Yan Q.; Hng H. H.; Zhang H. An Effective Method for the Fabrication of Few-Layer-Thick Inorganic Nanosheets. Angew. Chem., Int. Ed. 2012, 51, 9052–9056. 10.1002/anie.201204208. 22887481

[ref22] Zheng J.; Zhang H.; Dong S.; Liu Y.; Tai Nai C.; Suk Shin H.; Young Jeong H.; Liu B.; Ping Loh K. High yield exfoliation of two-dimensional chalcogenides using sodium naphthalenide. Nat. Commun. 2014, 5, 2995 10.1038/ncomms3995. 24384979

[ref23] Kong D.; Wang H.; Cha J. J.; Pasta M.; Koski K. J.; Yao J.; Cui Y. Synthesis of MoS_2_ and MoSe_2_ Films with Vertically Aligned Layers. Nano Lett. 2013, 13, 1341–1347. 10.1021/nl400258t. 23387444

[ref24] Zhang Y.; Yao Y.; Sendeku M. G.; Yin L.; Zhan X.; Wang F.; Wang Z.; He J. Recent Progress in CVD Growth of 2D Transition Metal Dichalcogenides and Related Heterostructures. Adv. Mater. 2019, 31, 1901694 10.1002/adma.201901694. 31402526

[ref25] Xia J.; Huang X.; Liu L.-Z.; Wang M.; Wang L.; Huang B.; Zhu D.-D.; Li J.-J.; Gu C.-Z.; Meng X.-M. CVD synthesis of large-area, highly crystalline MoSe_2_ atomic layers on diverse substrates and application to photodetectors. Nanoscale 2014, 6, 8949–8955. 10.1039/C4NR02311K. 24965908

[ref26] Varade V.; Haider G.; Pirker L.; Panda J.; Sykora J.; Frank O.; Kalbac M.; Vejpravova J. Sulfur isotope engineering of exciton and lattice dynamics in MoS_2_ monolayers. 2D Mater. 2023, 10, 025024 10.1088/2053-1583/acc4d8.

[ref27] Klapetek P.; Necas D. Gwyddion: an open-source software for SPM data analysis. Cent. Eur. J. Phys. 2012, 10, 181–188. 10.2478/s11534-011-0096-2.

[ref28] Verhagen T.; Guerra V. L. P.; Haider G.; Kalbac M.; Vejpravova J. Towards the evaluation of defects in MoS _2_ using cryogenic photoluminescence spectroscopy. Nanoscale 2020, 12, 3019–3028. 10.1039/C9NR07246B. 31834348

[ref29] Cardona M.; Thewalt M. L. W. Isotope effects on the optical spectra of semiconductors. Rev. Mod. Phys. 2005, 77, 1173–1224. 10.1103/RevModPhys.77.1173.

[ref30] Michail A.; Delikoukos N.; Parthenios J.; Galiotis C.; Papagelis K. Optical detection of strain and doping inhomogeneities in single layer MoS_2_. Appl. Phys. Lett. 2016, 108, 173102 10.1063/1.4948357.

[ref31] Placidi M.; Dimitrievska M.; Izquierdo-Roca V.; Fontané X.; Castellanos-Gomez A.; Pérez-Tomás A.; Mestres N.; Espindola-Rodriguez M.; López-Marino S.; Neuschitzer M.; et al. Multiwavelength excitation Raman scattering analysis of bulk and two-dimensional MoS_2_: Vibrational properties of atomically thin MoS_2_ layers. 2D Mater. 2015, 2, 035006 10.1088/2053-1583/2/3/035006.

[ref32] Gołasa K.; Grzeszczyk M.; Bożek R.; Leszczyński P.; Wysmołek A.; Potemski M.; Babiński A. Resonant Raman scattering in MoS_2_—From bulk to monolayer. Solid State Commun. 2014, 197, 53–56. 10.1016/j.ssc.2014.08.009.

[ref33] Chakraborty B.; Matte H. S. S. R.; Sood A. K.; Rao C. N. R. Layer-dependent resonant Raman scattering of a few layer MoS_2_. J. Raman Spectrosc. 2013, 44, 92–96. 10.1002/jrs.4147.

[ref34] Haider G.; Lin H.-I.; Yadav K.; Shen K.-C.; Liao Y.-M.; Hu H.-W.; Roy P. K.; Bera K. P.; Lin K.-H.; Lee H.-M.; et al. A Highly-Efficient Single Segment White Random Laser. ACS Nano 2018, 12, 11847–11859. 10.1021/acsnano.8b03035. 30352157

[ref35] Zhang X.; Zhang R.; Zhang Y.; Jiang T.; Deng C.; Zhang X.; Qin S. Tunable photoluminescence of bilayer MoS_2_ via interlayer twist. Opt. Mater. 2019, 94, 213–216. 10.1016/j.optmat.2019.05.047.

[ref36] Kalbac M.; Farhat H.; Kong J.; Janda P.; Kavan L.; Dresselhaus M. S. Raman Spectroscopy and in Situ Raman Spectroelectrochemistry of Bilayer ^12^C/^13^C Graphene. Nano Lett. 2011, 11, 1957–1963. 10.1021/nl2001956. 21506590

[ref37] Kalbac M.; Lehtinen O.; Krasheninnikov A. V.; Keinonen J. Ion-Irradiation-Induced Defects in Isotopically-Labeled Two Layered Graphene: Enhanced In-Situ Annealing of the Damage. Adv. Mater. 2013, 25, 1004–1009. 10.1002/adma.201203807. 23180424

