# Supplementary material for: Addressing Individual Layers and Their Optical Properties in Artificial MoS2 Bilayers via Sulfur Isotope Labeling
Source: J Phys Chem C Nanomater Interfaces. 2024 Jul 23;128(30):12575–81. doi: 10.1021/acs.jpcc.4c03132 (PMC11299176; doi:10.1021/acs.jpcc.4c03132)
Supplement: Supplementary file 1 — jp4c03132_si_001.pdf [file jp4c03132_si_001.pdf]

Supplementary Information

Addressing individual layers and their optical properties in artificial MoS_2 bilayer via Sulphur isotope labeling

Antonin Kralik,[1] Golam Haider,[1] Vaibhav Varade[2], Martin Kalbac[1], Jana Vejpravova**[2]*

[1]Heyrovsky Institute of Physical Chemistry of the CAS, v.v.i., Dolejskova 3, CZ-182 23 Prague 8, Czech Republic.

[2]Department of Condensed Matter Physics, Faculty of Mathematics and Physics, Charles University, Ke Karlovu 5, CZ-121 16 Prague 2, Czech Republic.

*kalbac@jh-inst.cas.cz

**jana.vejpravova@matfyz.cuni.cz

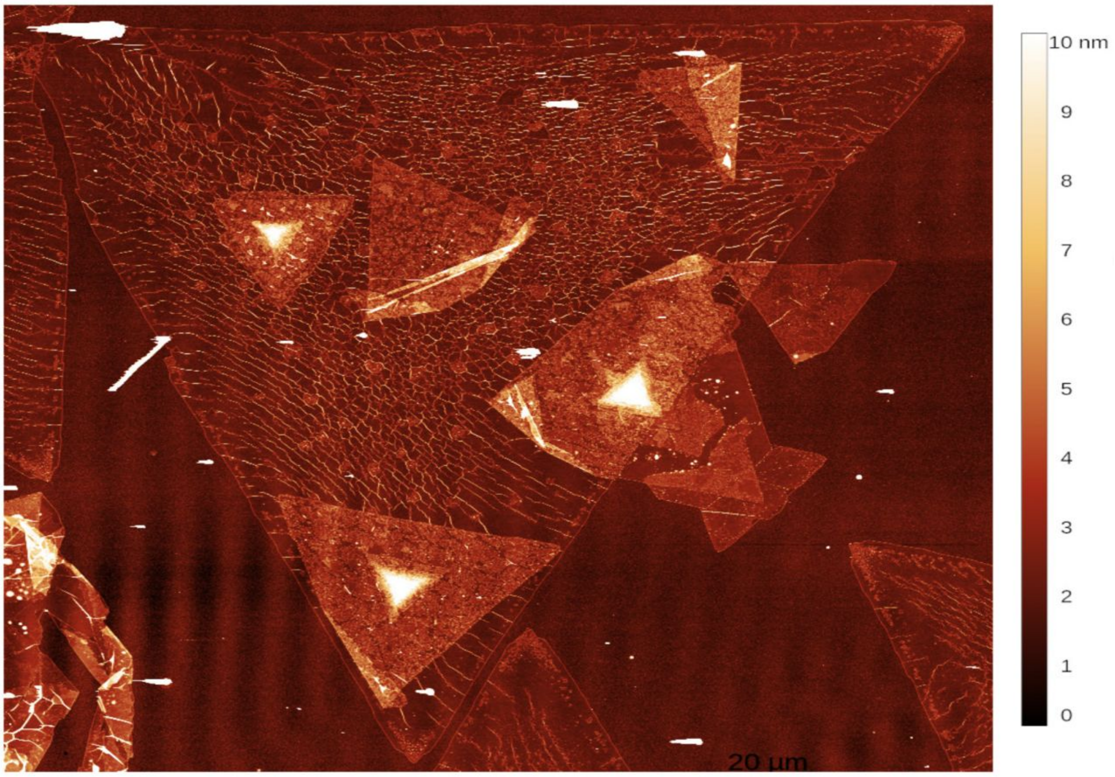

Figure S1. AFM image of the $Mo^{34}S_2/Mo^{Nat}S_2$ bilayer.

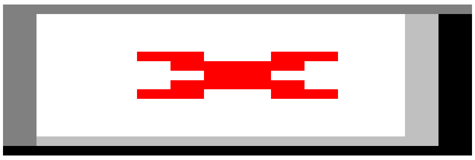

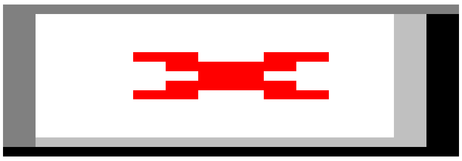

Figure S2. Raman spectroscopic characterization of $Mo^{Nat}S_2$ (a) and $Mo^{34}S_2$ (b) monolayers obtained using 633 nm laser excitation energy.

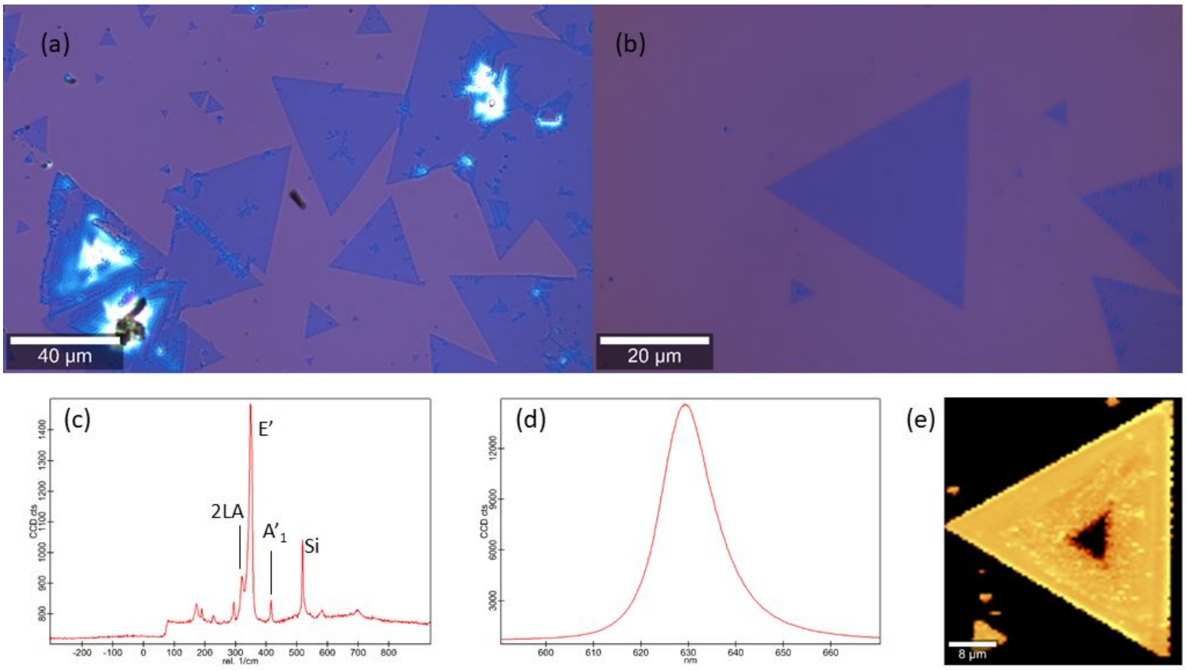

Figure S3. Optical images (a, b) and Raman € and PL (d) spectroscopic characterization of $W^{34}S_2$. Data recorded from the flake shown in panel (b). Spatial distribution of the PL peak full width at half maximum is given in panel €. Quenching of the PL in the flake center is very common in CVD-grown TMDs and it is due to add-layer.
